# Synthesis and Antidiabetic Evaluation of Benzenesulfonamide Derivatives

**Published:** 2013

**Authors:** Nouraddin Hosseinzadeh, Soodeh Seraj, Mohamad Ebrahim Bakhshi-Dezffoli, Mohammad Hasani, Mehdi Khoshneviszadeh, Saeed Fallah-Bonekohal, Mohammad Abdollahi, Alireza Foroumadi, Abbas Shafiee

**Affiliations:** a *Department of Medicinal Chemistry, Faculty of Pharmacy and Pharmaceutical Sciences Research Center, Tehran University of Medical Sciences, Tehran14176, Iran.*; b *Islamic Azad University, Shahre Rey Branch.*; c *School of chemistry, College of Science, University of Tehran, Tehran Iran.*; d *Department of Toxicology and Pharmacology, Faculty of Pharmacy and Laboratory of Toxicology, Pharmaceutical Sciences Research Centre, Tehran University of Medical Sciences, Tehran 14155-6451, Iran. *; e *Drug Design and development Research Center, Tehran University of Medical Sciences, Tehran, Iran. *

**Keywords:** Thiazole, Benzenesulfonamides, Antidiabetic Activity, Diabetes Mellitus

## Abstract

The complex metabolic syndrome, diabetes mellitus, is a major human health concern in the world and is estimated to affect 300 million people by the year 2025. Several drugs such as sulfonylureas and biguanides are presently available to reduce hyperglycemia in diabetes mellitus. These drugs have side effects and thus searching for a new class of compounds is essential to overcome this problems.

A series of seven novel *N*-(4-phenylthiazol-2-yl)benzenesulfonamides derivatives were synthesized and assayed *in-vivo *to investigate their antidiabetic activities by streptozotocin-induced model in rat. These derivatives showed considerable biological efficacy when compared to glibenclamide, a potent and well-known antidiabetic agent, as a reference drug. Four of the compounds were

effective, amongst which 13 show more prominent activity at 100 mg/Kg p.o. The experimental results are statistically significant at p < 0.05 level.

## Introduction

Diabetes mellitus is a metabolic alteration characterized by hyperglycemia resulting from defects in insulin secretion, action, or both, currently affecting *ca*. 3% of the world population.

This complex metabolic syndrome is a major human health concern in the world and is estimated to affect 300 million people by the year 2025 (1, 2).

Most of the diabetic patients are known as non-insulin dependent diabetes mellitus (NIDDM). Resistance to the biological actions of insulin in the liver and peripheral tissues, together with pancreatic cell defects, is a major feature of the pathophysiology of human NIDDM (3, 4).

Pharmaceutical intervention of hyperglycemia induced diabetic complications is actively pursued since it is very difficult to maintain normoglycemia by any means in patients with diabetes mellitus (5, 6).

Several drugs such as sulfonylureas and biguanides are presently available to reduce hyperglycemia in diabetes mellitus. These drugs demonstrated significant side effects and thus searching for a new class of compounds is essential to overcome these problems (7). Therefore, the urgent need to look for novel drug scaffold with minimal side effects is still a challenge to the medicinal chemist (8).

The clinical and medicinal importance of sulfonamides is well documented. The sulfonamide moiety (–SO_2_NH_2_) is an active pharmacophore, exhibiting a wide variety of pharmacological activities such as antimicrobial, antimalarial, insulin-releasing antidiabetic, anti-HIV, high ceiling diuretic, antithyroid, and antitumor (9-12).

Among the broad spectrum of activities exhibited by sulfonamides, their role as antidiabetic is more considerable (13, 14).

In continuation of our research program to develop small molecules as biologically active compounds (15-19), in this paper we report the synthesis and structural characterization of several benzenesulfonamides derivatives. These compounds were evaluated for their hypoglycemic activity after administration at dose of 100 mg/Kg in Alloxan-STZ induced diabetic rat. Blood glucose level were measured and compared with control drug, Glibenclamide (5 mg/Kg) as a standard. 

## Experimental

*Chemistry*

The target compounds were synthesized according to the two step reaction protocol. The general synthetic pathways are shown in Figure 1. 2-bromo-1-(4-methoxyphenyl)ethanone (1) was reacted with thiourea in refluxing ethanol to yield 4-(4-methoxyphenyl)thiazol-2-amine (3, R = MeO). In addition 4-(4-chlorophenyl) thiazol-2-amine (4, R = Cl) was produced through the reaction of 1-(4-chlorophenyl) ethanone (2) with thiourea in the presence of iodine in refluxing ethanol (20).

The target compounds were synthesized by simple and facile condensation reaction of equimolar quantities of 2-amino thiazol (compounds 3, 4) with appropriate sulfonyl chloride (compounds 5-11). The reactions were stirred at room temperature in pyridine for 4 days. The solid products was obtained by filtration and purified by recrystallization.

The synthesized compounds 12-19 were characterized by ^1^H NMR, IR and Mass spectroscopy. The hydrogen of amine in compounds 12-19 was detected at 8.6-9.0 ppm as a broad peak which was deshielded by an adjacent sulfonyl group. The feature of the benzenesulfonamides in the solid state is also supported by the IR spectral data (NH group band at ~ 3300 cm^-1^ and S=O band at ~ 1281-1157 cm^-1^) for the majority of the compounds.

*Synthesis of 4-(4-methoxyphenyl) thiazol-2-amine (3)*

The experimental protocol is based on a previously described methodology (20). To a solution of 2- boromo-1-(4-methoxyphenyl) ethanone (228 mg, 1 mmol) in 5 mL of ethanol, a solution of thiourea (76 mg, 1 mmol) in 10 mL of ethanol was added. The mixture was refluxed for 1.5 h. The solution was neutralized with ammonia and the precipitate was filtered, washed with water and the product was purified by recrystallization from diethyl ether.

*Synthesis of 4-(4-chlorophenyl) thiazol-2-amine (4)*

The mixture of thiourea (76 mg, 1 mmol) and iodine (253.8 mg, 1 mmol) in 10 mL of ethanol was added to the solution of 1-(4-chlorophenyl) ethanone (154 mg, 1 mmol) in 5 mL of ethanol. The mixture was heated under reflux for 1 h and stirred at room temperature for 24 h.

After cooling, the precipitate was filtered, washed with water and the resulted crude product was purified by recrystallization from diethyl ether (20).

*General procedure for the synthesis of N-(4-(4-methoxyphenyl or 4-chlorophenyl)thiazol-2-yl) benzenesulfonamid (12-19)*

A mixture of 4-(4-methoxyphenyl or 4-chlorophenyl) thiazol-2-amine (1 mmol) and appropriate sulfonyl chloride (1 mmol) in pyridine (2 mL) was stirred at room temperature for 4 days. The mixture was evaporated under reduced pressure and the mixture was neutralized with dilute hydrochloric acid. The precipitate was filtered and washed with water and the resulting crude product was purified by recrystallization from methanol (20).

*N-(4-(4-Methoxyphenyl)thiazol-2-yl)benzenesulfonamid (12)*

Yield: 53 %; mp: 258-260°C; IR (KBr, cm^-1^): 3289 (NH), 1173 and 1255 (S=O), 1646 (C=N). ^1^H NMR (DMSO-*d*_6_) δ: 3.80 (s, 3H), 7.04 (d, 2H, *J *= 8. 7 Hz), 7.09 (s, 1H), 7.73 (d, 2H, *J *= 8.7 Hz), 8.06 (t, 1H, *J *= 7.7 Hz), 8.59 (t, 1H, *J *= 7.7 Hz), 8.9 (s, 1H, NH), 8.92 (d, 2H, *J *= 7.7 Hz). MS: m/z (%) 346 (M^+^, 1.5), 206 (100), 191 (30), 164 (11), 149 (27), 94 (22), 77 (30). Anal. Calcd for C_16_H_14_N_2_O_3_S_2_: C, 55.47; H, 4.07; N, 8.09. Found: C, 55.26; H, 3.79; N, 7.79.

*2, 5-Dichloro-N-(4-(4-methoxyphenyl) thiazol-2-yl)benzenesulfonamide (13)*

Yield: 43 %; mp: 233-235°C; IR (KBr, cm^-1^): 3302 (NH), 1148 and 1296 (S=O), 1638 (C=N). ^1^H NMR (DMSO-*d*_6_) δ: 3.79 (s, 3H), 7.04 (d, 2H, *J *= 8.2 Hz ), 7.07 (s,1H), 7.41 (m, 2H), 7.64 (d, 2H, *J *= 8.2), 7.84 (s, 1H), 8.9 (s, 1H, NH). MS: m/z (%) 414 (M ^+^, 0.1), 236 (4), 226 (13), 206 (100), 191 (16), 164 (10), 149 (14), 109 (5), 77 (5). Anal. Calcd for C_16_H_12_Cl_2_N_2_O_3_S_2_: C, 46.27; H, 2.91; N, 6.75. Found: C, 45.99; H, 2.60; N, 6.45.

*2, 4, 5-Trichloro-N-(4-(4-methoxyphenyl) thiazol-2-yl)benzenesulfonamide (14)*

Yield: 39 %; mp: 213-215°C; IR (KBr, cm^-1^): 3370 (NH), 1118 and 1322 (S=O), 1653 (C=N). ^1^H NMR (DMSO-*d*_6_) δ: 3.79 (s, 3H), 7.04 (d, 2H, *J *= 8.8 Hz), 7.06 (s, 1H), 7.64 (d, 2H, *J *= 8.8 Hz), 7.76 (s, 1H), 7.96 (s, 1H), 8.8 (s, 1H, NH). MS: m/z (%) 450 (M^+^+2, 0.7), 448 (M ^+^, 0.7 ), 238 (19), 206 (100), 191 (29), 171 (9), 164 (12), 149 (57), 121 (12), 107 (5). Anal. Calcd for C_16_H_11_Cl_3_N_2_O_3_S_2_: C, 42.73; H, 2.47; N, 6.23. Found: C, 42.42; H, 2.18; N, 5.94.

*4-Methoxy–N-(4-(4-methoxyphenyl)thiazol-2-yl)benzenesulfonamide (15)*

Yield: 48 %; mp: 233-235°C; IR (KBr, cm^-1^): 3326 (NH), 1187 and 1302 (S=O), 1640 (C=N). ^1^H NMR (DMSO-*d*_6_) δ: 3.74 (s, 3H), 3.79 (s, 3H), 6.86 (d, 2H, *J *= 8.4Hz), 7.03 (d, 2H, *J *= 8.6Hz), 7.05 (s, 1H), 7.53 (d, 2H, *J *= 8.4Hz), 7.65 (d, 2H, *J *= 8.6Hz), 8.8 (s, 1H, NH). MS: m/z (%) 376(M^+^, 4), 238 (17), 206 (100), 191 (29), 188 (21), 164 (12), 149 (23), 123 (12), 77 (13).Anal. Calcd for C_17_H_16_N_2_O_4_S_2_: C, 54.24; H, 4.28; N, 7.44. Found: C, 53.96; H, 4.00; N, 7.13.

*4-Bromo–N- (4-(4-methoxyphenyl) thiazol-2-yl)benzenesulfonamide (16)*

Yield: 61 %; mp: 250-252°C; IR (KBr, cm^-1^): 3289 (NH),1119 and 1302 (S=O), 1651 (C=N). ^1^H NMR (DMSO-*d*_6_) δ: 3.74 (s, 3H), 6.95(s, 1H), 6.99 (d, 2H, *J *= 8.6 Hz), 7.52 (m, 4H), 7.56 (d, 2H, *J *= 8.6 Hz), 8.7 (s, 1H, NH). MS: m/z (%) 426 (M^+^+2, 3), 424 (M^+^, 4), 238 (10), 206 (100), 191 (29), 164 (11), 149 (23), 121 (11), 77 (7). Anal. Calcd for C_16_H_13_BrN_2_O_3_S_2_: C, 45.18; H, 3.08; N, 6.59. Found: C, 44.87; H, 2.80; N, 6.30.

*N-(4-(4-Methoxyphenyl)thiazol-2-yl)-4-methylbenzenesulfonamide (17)*

Yield: 44 %; mp: 209-212°C; IR (KBr, cm^-1^): 3268 (NH), 1178 and 1244 (S=O), 1625 (C=N). ^1^H NMR (DMSO-*d*_6_) δ: 2.29 (s, 3H), 3.80 (s, 3H), 7.03 (d, 2H, *J *=8.7 Hz), 7.04 (s, 1H), 7.12 (d, 2H, *J *= 7.85 Hz), 7.48 (d, 2H, *J *= 7.85 Hz ), 7.68 (d, 2H, *J *= 8.7 Hz), 8.6 (s, 1H, NH). MS: m/z (%) 360(M^+^, 0.8), 206 (100), 191 (28), 164 (12), 149 (24), 121 (12), 77 (7). Anal. Calcd for C_17_H_16_N_2_O_3_S_2_: C, 56.65; H, 4.47; N, 7.77. Found: C, 56.35; H, 4.16; N, 7.47.

*4-Chloro–N-(4-(4-methoxyphenyl)thiazol-2-yl)benzenesulfonamide (18)*

Yield: 50 %; mp: 224-226°C; IR (KBr, cm^-1^): 3292 (NH), 1169 and 1299 (S=O), 1645 (C=N). ^1^H NMR (DMSO-*d*_6_) δ: 3.79 (s, 3H), 7.03 (d, 2H, *J *=8.75 Hz), 7.06 (s, 1H), 7.38 (d, 2H, *J *= 8.4 Hz), 7.61 (d, 2H, *J *= 8.4 Hz ), 7.64 (d, 2H, *J *= 8.75 Hz). MS: m/z (%) 382 (M^+^+2, 0.3), 380(M^+^, 0.8), 206 (100), 191 (29), 164 (10), 149 (24), 128 (15), 111 (9), 75 (10). Anal. Calcd for C_16_H_13_ClN_2_O_3_S_2_: C, 50.46; H, 3.44; N, 7.36. Found: C, 50.16; H, 3.13; N, 7.07.

*N-(4-(4-Chlorophenyl)thiazol-2-yl)-4-methylbenzenesulfonamide (19)*

Yield: 47%; mp: 288-290°C; IR (KBr, cm^-1^): 3330 (NH), 1165 and 1228 (S=O), 1644 (C=N). ^1^H NMR (DMSO-*d*_6_) δ: 2.28 (s, 3H), 7.11 (d, 2H, *J *=7.8Hz), 7.21 (s, 1H), 7.48 (d, 2H, *J *= 7.8 Hz), 7.51 (d, 2H, *J *= 8.4Hz ), 7.75 (d, 2H, *J *= 8.4 Hz ), 8.6 (s, 1H, NH). MS: m/z (%) 366 (M^+^+2, 0.15), 364 (M^+^, 0.4), 292 (7), 210 (100), 172 (32), 168 (51), 91 (68). Anal. Calcd for C_16_H_13_ClN_2_O_2_S_2_: C, 52.67; H, 3.59; N, 7.68. Found: C, 52.36; H, 3.30; N, 7.39.

*General antidiabetic activity procedure*

Adult male Wistar rats weighting 200-250 g were housed under standard environmental conditions of temperature (25 ± 2°C) and a 12 h light/dark cycle in animal house of PSRC/TUMS. They were fed by normal laboratory chow and water. Acute diabetes was induced by intravenous administration of streptozotocin-alloxan (40 mg/Kg of each) dissolved in 0.05 M citrate buffer with pH of 4.5 to 24-h fast rats. Blood glucose changes were measured by a glucometer from the rats’ tail veins every hour post administration of diabetes. Synthesized compounds were administered at a single dose of 100 mg/Kg. Glibenclamide (5 mg/Kg) as the standard was administered orally 48 h post administration of streptozotocin-alloxan. The basis of comparison was the blood glucose level 2 h post administration of drugs to diabetic rats.

## Results and Discussion

A series of N-(4-phenylthiazol-2-yl) benzenesulfonamides derivatives were synthesized by reacting of equimolar quantities of 2-amino thiazol (compound 3 and 4) with appropriate aromatic sulfonyl chloride. The structures of these compounds were established by means of IR, ^1^H NMR, and elemental analysis. All the compounds were screened *in-vivo *for their oral hypoglycemic activity by streptozotocin-induced diabetic model in rat. Four of the compounds demonstrated remarkable hypoglycemic property, however with a degree of variation. The results of changes in blood glucose in diabetic rats treated with 100 mg/Kg p.o. of the synthesized benzenesulfonamides derivatives are presented in (Table 1). A significant increase in blood glucose was observed in diabetic rats (control group). Compounds 12 and 13 showed significant reduction in blood glucose as compared to control diabetic rats at dose of 100 mg/Kg p.o. Glibenclamide was taken as standard drug which showed 32.7% blood glucose lowering activity at the dose of 5 mg/Kg p.o.

**Table 1:** Blood glucose reduction of synthesized compounds by Alloxan-STZ (40 mg/Kg of it IV injection) method in rat.

**Compound**	**Befor administration mean ± SEM**	**2 h post administration mean ± SEM**	**Reduction (%)** ^1^	**p-value**
^2^ Control (diabetic)	359 ± 4.5	354 ± 7.3	1.4	p < 0.05 vs. Glb. (**)
Glibenclamide	324 ± 6.8	218 ± 11.1	32.7	p < 0.05 vs. Control (***)
12	382 ± 6.5	303 ± 6.6	20.7	p < 0.05 vs. Control (*); Glb.
13	472 ± 7	343 ± 6.4	27.5	p < 0.05 vs. Control (**); Glb.
15	158 ± 9.5	179 ± 4.5	^3^-13.2	p < 0.05 vs. Control ; Glb.
16	173 ± 8.7	196 ± 3.4	^3^-13.2	p < 0.05 vs. Control; Glb.
17	382 ± 7.2	362 ± 13	5.2	p < 0.05 vs. Control; Glb. (***)
18	143 ± 6.4	155 ± 6.5	^3^-9.09	p < 0.05 vs. Control ; Glb.
19	275 ± 5.4	253 ± 9.8	7.6	p < 0.05 vs. Control ; Glb. (***)

^1^ % reduction obtained by (2-0 h)/2 h.100; ^2^Control group was diabetic only; Compounds administered at 100 mg/Kg and Glb. at 5 mg/Kg (solved in water by oral administration); ^3^Negative reduction means blood glucose is increased under the influence of drugs. ^***^ : Significant p < 0.001; ^**^ Significant P< 0.01; ^*^Significant p < 0.05

The results indicated that the introduction of 2,5-dichloro group on phenyl sulfonyl group potentiated the antidiabetic activity of studied compounds. On the other hand, replacement of para hydrogen atom of phenyl sulfonyl group with other substitutes resulted in reduced antidiabetic potential of benzensulfanamide derivatives. It could be deduced that the presence of any group at 4-position of the phenylsulfonyl might sterically hinder the effective interaction of studied compounds with their receptor.

## Conclusion

This study reports the synthesis and antidiabetic activity of novel *N*-(4-phenylthiazol-2-yl) benzenesulfonamides derivatives. Some of the synthesized compounds showed hypoglycemic activity. These results indicated that benzenesulfonamide could be served as potential antidiabetic agents in the same manner as sulfonylurea derivatives. It seems that structural modification of this scaffold will resulted in more potent oral antidibetic derivatives as it will be followed in our future projects.
